# Antigenotoxic and Apoptotic Activity of Green Tea Polyphenol Extracts on Hexavalent Chromium-Induced DNA Damage in Peripheral Blood of CD-1 Mice: Analysis with Differential Acridine Orange/Ethidium Bromide Staining

**DOI:** 10.1155/2013/486419

**Published:** 2013-12-02

**Authors:** María del Carmen García-Rodríguez, Megumi Monserrat Carvente-Juárez, Mario Agustín Altamirano-Lozano

**Affiliations:** Unidad de Investigación en Genética y Toxicología Ambiental (UNIGEN), Facultad de Estudios Superiores “Zaragoza”, Universidad Nacional Autónoma de México (UNAM), Batalla 5 de Mayo s/n, 09230, DF, Mexico

## Abstract

This study was conducted to investigate the modulating effects of green tea polyphenols on genotoxic damage and apoptotic activity induced by hexavalent chromium [Cr (VI)] in CD-1 mice. Animals were divided into the following groups: (i) injected with vehicle; (ii) treated with green tea polyphenols (30 mg/kg) via gavage; (iii) injected with CrO_3_ (20 mg/kg) intraperitoneally; (iv) treated with green tea polyphenols in addition to CrO_3_. Genotoxic damage was evaluated by examining micronucleated polychromatic erythrocytes (MN-PCEs) obtained from peripheral blood at 0, 24, 48, and 72 h after treatment. Induction of apoptosis and cell viability were assessed by differential acridine orange/ethidium bromide (AO/EB) staining. Treatment of green tea polyphenols led to no significant changes in the MN-PCEs. However, CrO_3_ treatment significantly increased MN-PCEs at 24 and 48 h after injection. Green tea polyphenols treatment prior to CrO_3_ injection led to a decrease in MN-PCEs compared to the group treated with CrO_3_ only. The average of apoptotic cells was increased at 48 h after treatment compared to control mice, suggesting that apoptosis could contribute to eliminate the DNA damaged cells induced by Cr (VI). Our findings support the proposed protective effects of green tea polyphenols against the genotoxic damage induced by Cr (VI).

## 1. Introduction

**Figure 1:**
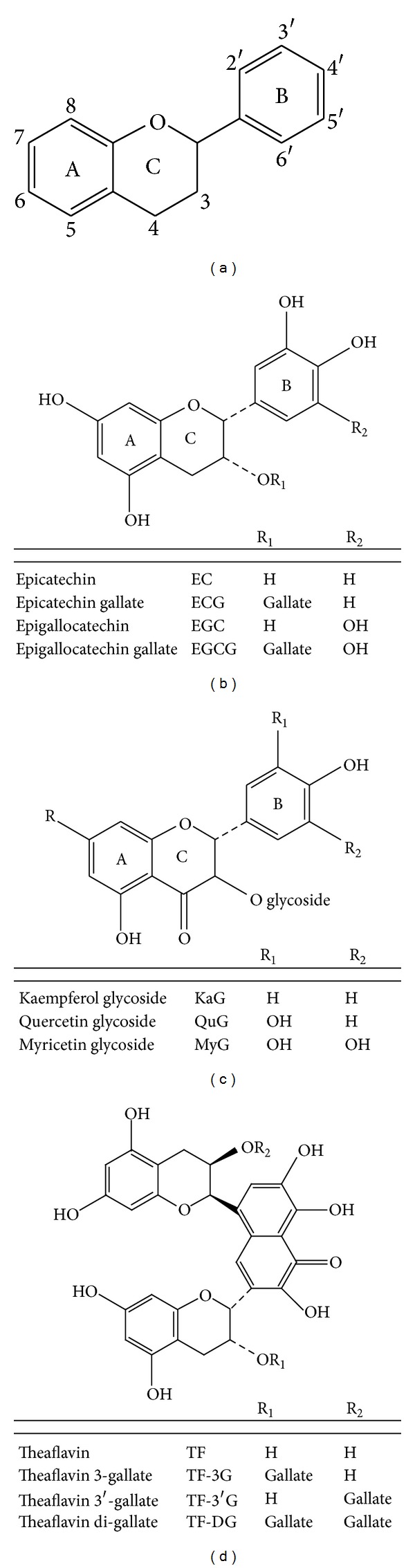
Classification of flavonoids. (a) Flavonoid diphenylpropane skeleton; (b) tea flavanols (flavan-3-ols); (c) tea flavonols; (d) tea theaflavins.

Green tea (*Camellia sinensis*) is one of the most ancient beverages, consumed by over two-thirds of the world's population. The principal constituents are caffeine, tannins, and essential oils. The tannins encompass a variety of polyphenolic compounds, including important flavonoids such as catechins: (−)-epicatechin (EC), (−)-epigallocatechin (EGC) and their gallate forms (+) gallocatechin (GC), (−) epicatechin-3-gallate (ECG) and (−) epigallocatechin-3-gallate (EGCG) [1, 2]. These compounds are chemically classified as dibenzpyrans, pyrones, and their derivatives. The core structure contains a diphenylpropane skeleton (Figure 1(a)). The primary flavonoids found in fresh green tea leaves are catechins (flavan-3-ols or flavanols) and the flavonols (Figures 1(b) and 1(c), resp.) [1]. In addition, green tea contains other polyphenols such as theaflavins (Figure 1(d)) but at lower concentration than catechins. The polyphenols are also naturally found in fruits and vegetables, as well as in drinks such as red wine and beer [1, 2].

Green tea has attracted significant attention recently, both in the scientific and in consumer communities for its health benefits for a variety of diseases associated with oxidative stress such as cancer, cardiovascular, and neurodegenerative diseases [3, 4]. The beneficial effects of green tea are attributed to the antioxidant properties of the polyphenolic compounds. In addition to the cancer chemopreventive properties, green tea polyphenols have shown anti-inflammatory, antiallergenic, antibacterial, and antiviral properties [4–6], as well as antimutagenic activity [7].

The polyphenol compounds have shown direct effects as radical scavengers and metal chelators and indirect effects via the modulation of transcription factors and enzymes [8, 9]. In fact, these antioxidants can inhibit the formation of 8-hydroxydeoxyguanosine (8-OH-dG, 7,8-dihydro-8-oxodeoxyguanosine) *in vivo* [10, 11]. Thus, substances with antioxidant properties have emerged as putative preventives and coadjuvants in the treatment of chronic degenerative diseases related to oxidative stress and DNA damage.

In contrast, hexavalent chromium [Cr (VI)] compounds are particularly effective at inducing genotoxicity by causing several types of DNA lesions and gene mutations. Cr (VI) compounds have been widely studied because they have various industrial applications, including chromium plating, metallurgy, pigment manufacturing, leather tanning, and wood preservation, and because they are associated with the induction of cancer [12]. Cr usually exists in various oxidation states, primarily Cr (III) and Cr (VI). Nonetheless, Cr (III) is an essential micronutrient and plays an important role in protein, sugar, and fat metabolism [13]. Cr (VI) is particularly effective at inducing genotoxicity by causing several types of DNA lesions and gene mutations. Cr (VI)-induced DNA-DNA interstrand cross-links, oxidative DNA damage, and mutations in the tumor suppressor gene *p53* are some of the major factors that may play a significant role in determining cellular genotoxicity [14, 15]. According to previous studies, Cr-induced genomic DNA damage includes 8-hydroxydeoxyguanosine (8-OH-dG, 7,8-dihydro-8-oxodeoxyguanosine), which is a form of oxidative DNA damage [16]. Maeng et al. [17] observed changes in 8-OH-dG levels in DNA when rats were exposed to Cr (VI) and suggest that DNA damage caused by Cr (VI) compounds may be partially associated with oxidative stress. Cr (VI) generates reactive oxygen species (ROS) and free radicals (FRs) via its intracellular reduction to Cr (III) via the Fenton and Haber-Weiss reaction [15, 18, 19]. Although the direct relationship between DNA-ROS and chromium-induced DNA damage is heavily debated and unclear, there have been several studies supporting the role of ROS in Cr (VI)-induced genotoxicity and cytotoxicity [20]. Moreover, it has been observed that Cr (VI) given orally to mice could induce dose- and time-dependent effects on hepatic oxidative stress and hepatocyte apoptosis [21]. Apoptosis is a process in which cell death is initiated and completed in an orderly manner through the activation and/or synthesis of gene products necessary for cell destruction [22]. Apoptosis plays a crucial role in a number of physiological and pathological processes and is accompanied by characteristic morphological changes, which include cytoplasmic shrinkage, plasma membrane blebbing, condensation or fragmentation of nuclei, and extensive degradation of chromosomal DNA. Indeed, many chemopreventive agents act by inducing apoptosis as a mechanism to suppress carcinogenesis [23]. Roy et al. [24] observed that EGCG not only protects normal cells against genotoxic alterations induced by MNNG but also eliminates cancer cells via the induction of apoptosis *in vitro*. We previously observed that *in vivo* administration of green tea (*ad libitum* for 10 days) decreased the induction of MN-PCEs upon treatment with CrO_3_. This result supports the protective effects of green tea against the genotoxic damage induced by metal compounds such as Cr (VI). However, the MN-PCEs induced by CrO_3_ were only partially blocked by the addition of green tea (approximately 42%) at days 1 and 2 [25]. This finding may be related to factors such as the origin of the tea because it has been observed that the amount of polyphenols in tea plants is influenced by environmental factors (i.e., weather, light, nutrients, preparation process, storage, horticulture leaf age, etc.). Also, it has been reported that polyphenols make up more than 30% of the dry weight of tea leaves; 90% of these compounds are catechins, and 10% are flavonols [26–28]. Therefore, as part of our research program that evaluates chemopreventive and chemoprotective components in the diet, to obtain a more efficient modulation of the genotoxic damage induced by Cr (VI) *in vivo*, we directly studied green tea polyphenol extracts that contain a mixture of polyphenolic compounds (minimum 60% total catechins with higher antioxidant activity [Polyphenon 60]), and we analyzed its apoptotic activity in the peripheral blood of CD-1 mice using analysis with differential acridine orange/ethidium bromide staining.

## 2. Materials and Methods

### 2.1. Chemicals

The following test chemicals and reagents were obtained from Sigma Chemicals Co. (St. Louis, MO, USA): CrO_3_ [CAS no. 1333-82-0], acridine orange (AO) [CAS no. 10127-02-3], ethidium bromide (EB) [CAS no. 1239-45-8], and green tea polyphenol extracts (Polyphenon 60) [CAS no. 138988-88-2].

### 2.2. Animals

Two- to three-month-old CD-1 male mice (28–35 g) were used in the experiments. The animals were kept under controlled temperature (22°C) with a 12-12 h light-dark period (light 07:00–19:00 h). Mice had free access to food (Purina-México chow for small rodents) and water. All of the mice were obtained from Harlan at “Facultad de Química, Universidad Nacional Autónoma de México” (UNAM) and were acclimated for a two-week period. The Bioethics Committee of the “Facultad de Estudios Superiores-Zaragoza”, UNAM approved the experimental protocols used in this study.

### 2.3. Experimental Design

The dosage of green tea polyphenols extract (Polyphenon 60) was based on results obtained in previous studies, which utilized other commercially available Polyphenon 60 (0.625–1.25% body weight) [29] and our preliminary studies to determine the maximum tolerated dose (MTD) that did not induce MN-PCEs. The CrO_3_ dose was selected according to previous studies that intraperitoneally (i.p.) administered 20 mg/kg. This CrO_3_ dosage induced MN-PCEs in the peripheral blood of mice [30].

The green tea polyphenol extracts and CrO_3_ were prepared in solution by dissolving the dry compounds in sterile distilled water. Following preparation of the compounds, the solutions (0.25 mL) were administered immediately. The control group was treated in an identical manner with vehicle only. The evaluation criteria and work conditions were set up according to the OECD guideline (474), Food and Drug Administration (FDA) guidelines, Environmental Protection Agency (EPA) guidelines, and guidelines for the testing of chemicals specified by the Collaborative Study Group for the Micronucleus Test (CSGMT) and the Mammalian Mutagenesis Study Group of the Environmental Society of Japan (JEMS.MMS) for the short-term mouse peripheral blood micronucleus test [31–35].

After establishing treatment doses, the effects of green tea polyphenol extracts on genotoxic damage in CrO_3_-treated mice were evaluated. This assessment was performed using MN-PCEs kinetic analysis [31]. Mice were assigned at random to one of the following groups (*n* = 5 mice per group).

1. Animals injected with vehicle (control group).
2. Animals treated with green tea polyphenol extract (30 mg/kg) by gavage.
3. Animals injected with CrO_3_ (20 mg/kg).
4. Animals treated with green tea polyphenol extract (30 mg/kg) by gavage and then (4 h later) injected with CrO_3_ (20 mg/kg).

### 2.4. Micronucleus Assay

Slides were covered with AO and prepared according to the technique described by Hayashi et al. [36]. Briefly, AO was dissolved in distilled water at a concentration of 1 mg/mL, and 10 *μ*L of this solution was placed on a preheated (approximately 70°C) clean glass slide. The AO was spread evenly on the slide by moving a glass rod back and forth over the slide, which was then air-dried. The AO-coated glass slides were stored in a dark, dry location at room temperature prior to experimental use.

To evaluate MN after treatment, 5 *μ*L peripheral blood samples were collected by piercing a tail blood vessel of the mice every 24 h during a four-day period (0 to 72 h). The samples were placed directly on slides previously treated with AO according to Hayashi et al. [36]. After the sample was placed on the slide, a coverslip (24 × 50 mm) was immediately placed on the slide, and its edges were sealed with rubber cement. All of the slide preparations were kept in plastic boxes in the dark at 4°C. These slide preparations cannot be stored permanently, but they can be stored for several days in a refrigerator if the coverslip has been sealed. Two slides were prepared for each mouse, and analysis of the slides was conducted after 12 h.

**Figure 2:**
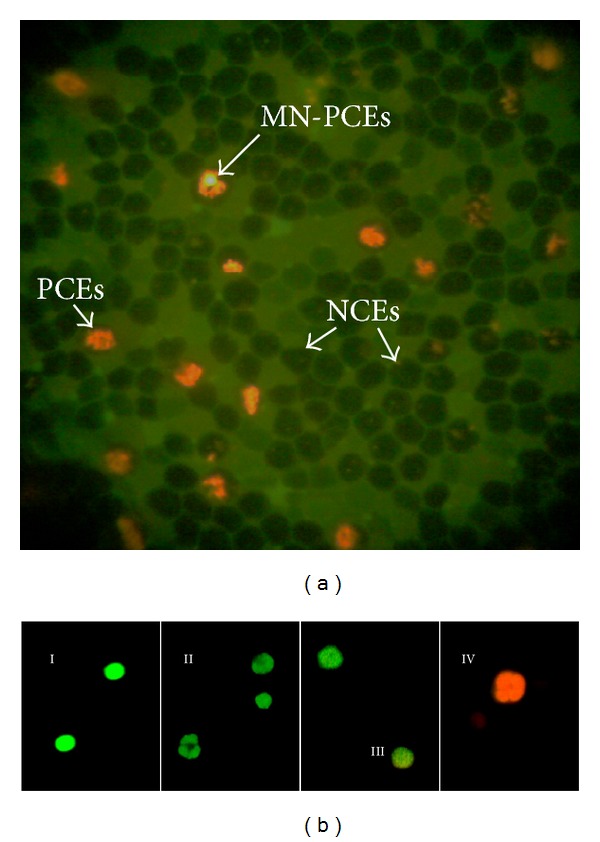
(a) Fluorescent microphotograph of CD-1 mouse peripheral blood cells using the AO coating method (NCEs, PCEs, and MN-PCEs). (b) Morphology of viable cells (apoptotic and nonapoptotic cells) and nonviable cells (apoptotic and nonapoptotic cells) as assessed by acridine orange/ethidium bromide staining. Viable cells stain uniformly green (I), early apoptotic cells with intact plasma membranes appear green, with “dots” of condensed chromatin that are highly visible within (II), late apoptotic cells are stained bright green-orange because membrane blebbing starts to occur, EB can enter the cell (III), and apoptotic nonviable cells are stained bright orange because of the entry of ethidium bromide into these cells (VI).

PCEs, NCEs, and MN-PCEs were identified under a fluorescent microscope (Nikon OPTIPHOT-2) using a blue excitation filter and a yellow barrier filter. The differential AO staining distinguished PCEs from NCEs, as PCEs were stained a fluorescent red-orange color due to the presence of ribosomal RNA. The AO staining also identified MN-PCEs, which were stained a fluorescent green color due to their DNA content (Figure 2(a)). The MN-PCEs analysis was based on the results from 2,000 cells per mouse, and the presence of MN-PCEs was used as a marker for genotoxic damage [36].

### 2.5. Apoptosis and Cell Viability Analyses

To evaluate apoptosis and cell viability we used the differential acridine orange/ethidium bromide (AO/EB) staining. Blood samples (100 *μ*L) were collected by piercing a tail blood vessel of the mice prior to treatment and 48 h after treatment. Heparin (10 *μ*L) was added to the blood samples, and 20 *μ*L of AO/EB dye mix (100 *μ*L/mL AO and 100 *μ*L/mL EB, both prepared in PBS) was then added. The suspension was concentrated via centrifugation (5,000 rpm), and the cell pellet was resuspended in 10 *μ*L and plated on a clean slide; a coverslip (24 × 24 mm) was immediately placed on the slide. Two slides were prepared per mouse, and the analysis was conducted immediately.

Apoptosis was assessed by identifying apoptotic, viable and nonviable cells under a fluorescent microscope (Nikon OPTIPHOT-2) with a blue excitation (480 nm) and a barrier filter (515–530 nm). The differential AO/EB staining is capable of distinguishing between viable and nonviable cells based on membrane integrity. When the cell is viable, the AO intercalates into the DNA, giving the cell a green appearance. Conversely, when the cell is nonviable, the EB also intercalates into the DNA, making the cell appear orange. Thus, a nonviable cell will contain a bright orange nucleus as EB overwhelms AO staining. Both healthy and apoptotic nuclei in viable cells will fluoresce bright green. In contrast, healthy or apoptotic nuclei in nonviable cells will fluoresce bright orange [33]. The apoptotic and cell viability assessments were based on 200 cells per mouse.

### 2.6. Statistical Analysis

The MN-PCEs induction results and the viable cells (apoptotic and nonapoptotic) and nonviable cells (apoptotic and nonapoptotic) data are expressed as the mean ± standard deviation (S.D.), and results from the various treatment groups were compared by an ANOVA test followed by a Tukey test. The net induction frequency (NIF) of MN-PCEs was analyzed using a Chi-square test [30, 37]. SPSS/PC V18TM and Statistica/PC V 6.0TM software were used for the statistical analyses. For all of the analyses, *P* < 0.05 was considered to be significant.

## 3. Results

The results obtained in the present study are shown in Table 1. Vehicle and green tea polyphenol extract did not modify the average number of induced MN-PCEs in the treated mice. CrO_3_ treatment increased the average number of MN-PCEs in all of the samples, but statistical significance was only observed at the 48 h time point when compared to the 0 h samples and the control group. Furthermore, CrO_3_ treatment increased the average number of MN-PCEs (approximately 13 MN-PCEs) compared to vehicle-treated mice. When the treatment included both green tea polyphenol extract and CrO_3_, we observed a decrease in the average number of MN-PCEs at 24, 48, and 72 h after treatment when compared to MN-PCEs induction in the CrO_3_ only treatment group, but the MN-PCEs induction observed at 48 remains statistically significant compared with the control group (Table 1). To compare the kinetics of MN-PCE induction in the various treatment groups, the data were analyzed by calculating the NIF value, which was calculated as follows [30]:

(1) NIF=number  of  MN-PCEs  measured  at  time  xi −number  of  MN-PCEs  measured  at  time  0,

where *x*_*i*_ = evaluation at 24, 48, or 72 h. Time 0 = evaluation at 0 h (before treatment).

**Figure 3:**
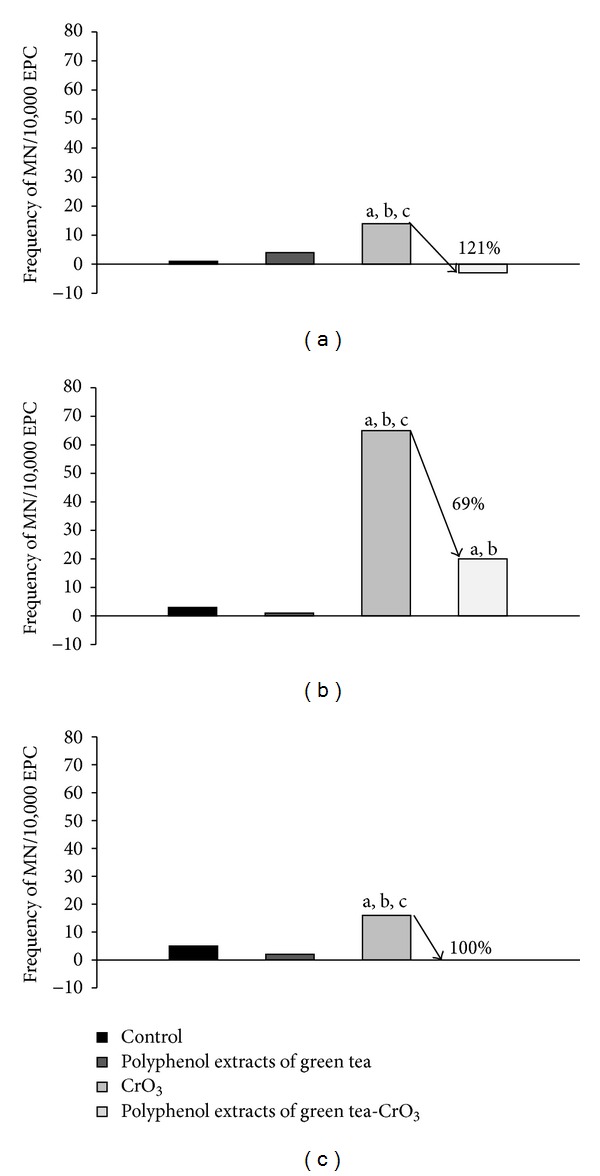
Effects of polyphenol extracts of green tea on the MN-PCEs frequency (% reduction) in mice peripheral blood at different times: (a) 24 h, (b) 48 h, and (c) 72 h after treatment with CrO_3_. Data represent MN-PCEs frequency obtained at 24, 48, and 72 h minus the MN-PCEs frequency at 0 h (NIF). ^a^ *P* < 0.05  versus control group; ^b^ *P* < 0.05  versus polyphenol extracts of green tea group; ^c^ *P* < 0.05  versus CrO_3_-polyphenol extracts of green tea group. *n* = 5 (2000 cells for mouse).

When the NIF is calculated, the net MN-PCEs induction can be more readily observed. This calculation subtracts the frequency of MN-PCEs prior to treatment from the frequency following treatment, thereby eliminating the baseline MN-PCEs variability that occurs between the treatment groups at time 0 (see S.D. Table 1).  Figure 3 presents the NIF values for all treatments at 24, 48, and 72 h after treatment. The groups treated with green tea polyphenol flavonoid extracts exhibited a principal MN-PCEs reduction at 24 and 72 h after treatment (approximately 121% and 100%, resp.).

Apoptosis and cell viability were evaluated directly in the peripheral blood of mice before (0 h) and after (48 h) treatment. Induction of apoptosis and cell viability were assessed via AO/EB staining of peripheral blood collected from treated mice. This technique shows the differential uptake of the fluorescent DNA-binding dyes AO and EB to determine viable and nonviable cells. These dyes were used to identify cells that have undergone apoptosis and to distinguish between cells in the early or late stages of apoptosis based on membrane integrity (Figure 2(b)). AO intercalates into the DNA, giving it a green appearance. This dye also binds to RNA, but because it cannot intercalate, the RNA stains red orange. Thus, a viable cell will have a bright green appearance. EB is only taken up by nonviable cells. This dye also intercalates into DNA, making it appear orange; however, EB only binds weakly to RNA, which may appear slightly red. Thus, a nonviable cell will have a bright orange nucleus, as EB overwhelms AO staining, and its cytoplasm will appear dark red (if any content remains). Both normal and apoptotic nuclei in viable cells will fluoresce bright green (Figure 2(b), I–III). In contrast, normal or apoptotic nuclei in nonviable cells will fluoresce bright orange (Figure 2(b), IV). Therefore, one can differentiate between early and late apoptotic cells using this system. Viable cells with intact membranes will have a uniformly stained green nucleus (Figure 2(b), I). Early apoptotic cells with intact membranes but that have started to fragment their DNA will still have green nuclei because EB cannot enter the cell, but chromatin condensation can be visualized as bright green patches in the nuclei (Figure 2(b), II). As the cell progresses through the apoptotic pathway and membrane blebbing starts to occur, EB can enter the cell, causing the cell to stain green orange (Figure 2, III). Late apoptotic cells will have bright orange patches of condensed chromatin in the nucleus that will distinguish these cells from necrotic cells, which will be uniformly stained orange (Figure 2, IV).

All of the treatments increased the average number of apoptotic viable cells, but statistical significance was achieved only in the CrO_3_ treatment group and the combined green tea polyphenol extract-CrO_3_ group compared to the control group. The increase in apoptosis was greater in the combined green tea polyphenol extract-CrO_3_ group than in the CrO_3_ group (12.8 versus 10.2 cells, resp.). The green tea polyphenol extract only treatment group had an increase of approximately four apoptotic viable cells compared to the control group. The average numbers of apoptotic nonviable cells and nonapoptotic cells were unchanged in all of the treatment groups (Table 2).

## 4. Discussion

The antioxidant properties present in polyphenols make them potentially useful for counteracting the DNA damage induced by oxidative stress agents such as Cr (VI) compounds. In this study, we evaluated the capability of polyphenol extracts from green tea to inhibit the genotoxic damage induced by Cr (VI) *in vivo* and analyzed apoptosis in peripheral blood of CD-1 mice.

The genotoxicity of Cr (VI) was demonstrated by the observation of a significant increase in MN-PCEs at 24 and 48 h after treatment in the CrO_3_-treated group (Table 1). This increase was clearly observed in all samples compared to the negative control, and this finding corroborates the previously reported genotoxicity of Cr (VI) and particularly CrO_3_ [15, 30, 38]. The mechanism of genotoxicity for Cr (VI) compounds has been linked to the intracellular reduction of Cr (VI) to Cr (III). Cr (VI) compounds can cross cell membranes via nonspecific anion transporters and are reduced by their interaction with intracellular cytoplasmic molecules. During this process, FRs are generated, which are capable of inducing genotoxic alterations [15, 18, 19]. Hence, we suggest that these effects can lead to the formation of MN-PCEs.

However, the average number of MN-PCEs revealed an increase of less than 1 MN-PCEs at 24 h after treatment in the group treated with green tea polyphenol extracts (Table 1), and the calculated NIF value corroborates the nongenotoxic effect of these extracts (Figure 3). It has been reported that the administration of polyphenol or flavonoids from green tea does not induce genotoxicity; additionally, the administration of these compounds over a long period of time at high doses in experimental animals has no effect on genotoxicity [4, 39, 40].

The *in vivo* administration of green tea polyphenol extracts prior to CrO_3_ injection decreased MN-PCEs formation by 121%, 69% and 100% at 24, 48, and 72 h after treatment, respectively, compared to MN-PCEs formation in the group treated with CrO_3_ alone (Figure 3). This result demonstrates that green tea polyphenol extracts protected cells against Cr (VI)-induced genetic damage more effectively than the administration of green tea *ad libitum* [25]. Due to the phenolic structure of green tea polyphenol extracts (Figure 1), it is possible that these flavonoids may act as hydrogen donors to suppress the formation of lipid radicals and FRs, including the superoxide and hydroxyl radicals generated by the Fenton reaction. These flavonoids may also chelate metals via their ortho-hydroxy-phenolic groups [41, 42]. Therefore, therapeutic agents that enhance intra- and extracellular antioxidant levels and block the Cr (VI)-mediated generation of ROS and FRs may prevent or attenuate Cr (VI)-induced genotoxicity.

In our experiments, the average number of apoptotic viable cells was increased at 48 h after treatment (Table 2). This increase was observed in all treatment groups compared to the negative control, and this finding supports the previously reported observations following Cr (VI) treatment [43–45]. The alteration of intracellular oxidative states has a potential to trigger or sensitize a cell to undergo apoptosis; thus, the ROS generated from Cr (VI) during its reduction plays an important role in the apoptotic signaling pathway [45, 46]. The administration of green tea polyphenol extracts alone led to an increase in the average number of apoptotic viable cells (approximately 4), but this increase was not statistically significant. Other studies have shown that green tea polyphenols such as EGCG and ECG inhibit the growth of the human lung cancer (cell line PC-9) [47]. The study further demonstrated that growth inhibition was accompanied by cell cycle arrest at the G2/M phase [48], which could be related to the apoptotic activity of these polyphenols. The induction of apoptosis by catechins has also been demonstrated in human lymphoid leukemia cells, human epidermoid carcinoma cells, human carcinoma keratinocytes, and human prostrate carcinoma cells [3, 49, 50]; thus, apoptosis plays an essential role as a protective mechanism against carcinogenesis by eliminating genetically damaged cells.

When the green tea polyphenol extracts were administered prior to the injection of CrO_3_, the average number of apoptotic viable cells was increased to a level that was higher than that observed following CrO_3_ treatment alone, but these interactions have shown that the effects are not additive or antagonistic (Table 2). Gao et al. [51] observed that ascorbic acid could enhance the EGCG and theaflavin-3-30-digallate induced apoptosis in human lung adenocarcinoma SPC-A-1 cells and esophageal carcinoma Eca-109 cells. Other studies have shown that the apoptosis-inducing activity of EGCG in human lung adenocarcinoma (PC-9 cells) can be synergistically enhanced by combined treatment with chemopreventive agents (including sulindac, cisplatin, and tamoxifen) [3, 52, 53]. The enhanced induction of apoptosis following a combined treatment suggests that this process may contribute to eliminate the cells with damaged DNA induced by Cr (VI).

In summary, the current study demonstrates that administration of CrO_3_ via i.p. injection of mice could induce DNA damage in peripheral blood and apoptosis, and these effects were time dependent. ROS and FRs formation may play an essential role in Cr (VI)-mediated DNA damage and apoptosis *in vivo*. Moreover, the polyphenol extracts derived from green tea are capable of reducing genotoxic damage induced by Cr (VI). The greatest degree of protection was observed at 24 h after injection of CrO_3._ The magnitude of protection is given in the following order: 121% at 24 h, >69% at 48 h, and >100% at 72 h after injection. Based on these results, polyphenol extracts from green tea can effectively protect against genotoxic damage in mice treated with Cr (VI). The beneficial effects of green tea polyphenol extracts could result from the inhibition of ROS and FRs chain reactions generated by the oxidative stress caused by Cr (VI) and by the extract's apoptotic activity.

There is limited evidence demonstrating that the regular consumption of green tea may reduce genotoxic damage. Therefore, this study contributes *in vivo* evidence showing that green tea polyphenol extracts can protect against genotoxic damage induced by carcinogens related to oxidative stress, such as Cr (VI) compounds.

## Figures and Tables

**Table 1:** Averages of the induction MN-PCEs in peripheral blood of mice treated with polyphenol extracts of green tea and CrO_3_.

Treatment	Dose (mg/kg)	*n*	Time analysis (hours)	MN-PCEs 2,000 cells (mean ± S.D.)	ANOVA
Control	0	5	0	0.6 ± 0.9	
			24	0.8 ± 0.8	
			48	1.2 ± 0.8	
			72	1.6 ± 1.5	

Polyphenol extracts of green tea	30	5	0	1.0 ± 1.2	
			24	1.8 ± 1.9	
			48	1.2 ± 1.3	
			72	1.4 ± 0.5	

CrO_3_	20	5	0	0.2 ± 0.5	
			24	3.0 ± 1.6	
			48	13.2 ± 3.8	a, b, c, d
			72	3.4 ± 1.1	

Polyphenol extracts of green tea–CrO_3_	30–20	5	0	1.2 ± 1.3	
			24	0.6 ± 0.5	
			48	5.2 ± 1.8	a, b, e
			72	1.2 ± 1.1	

^a^ *P*< versuscontrol 48 h; ^b^ *P*< versus polyphenol extracts of green tea 48 h; ^c^ *P*< versus CrO_3_ 0 h; ^d^ *P*< versus polyphenol extracts of green tea–CrO_3_ 48 h; ^e^ *P*< versus polyphenol extracts of green tea–CrO_3_ 0 h.

**Table 2:** Averages of viable cells (apoptotic and nonapoptotic) and nonviable cells (apoptotic and nonapoptotic) in peripheral blood of mice treated with polyphenol extract of green tea and CrO_3_.

Treatment	Dose(mg/kg)	*n*	Nonapoptotic viable cells* (mean ± S.D.)	Apoptotic viable cells* (mean ± S.D.)	Apoptotic nonviable cells* (mean ± S.D.)	Nonapoptotic nonviable cells* (mean ± S.D.)
Control	0	5	197.2 ± 2.2	2.4 ± 1.8	0.0 ± 0.0	0.4 ± 0.9
Polyphenol extracts of green tea	30	5	192.8 ± 4.9	6.0 ± 4.1	0.8 ± 1.3	0.4 ± 0.6
CrO_3_	20	5	189.2 ± 3.3^a^	10.2 ± 3.6^c^	0.2 ± 0.5	0.4 ± 0.6
Polyphenol extracts of green tea–CrO_3_	30–20	5	187.2 ± 6.3^b^	12.8 ± 6.3^d^	0.0 ± 0.0	0.0 ± 0.0

^a^ *P* < 0.05 versus control; ^b^ *P* < 0.01 versus control; ^c^ *P* < 0.04 versus control; ^d^ *P* < 0.007 versus control.

*Evaluation in 200 cells.

## Abbreviations

AO: Acridine orange
Cr (VI): Hexavalent chromium
EB: Ethidium bromide
EC: Epicatechin
ECG: Epicatechin-3-gallate
EGC: Epigallocatechin
EGCG: (−)-epigallocatechin-3-gallate
FDA: Food and Drug Administration
FRs: Free radicals
GC: (+) gallocatechin
i.p.: Intraperitoneal
MNNG: N-methyl-N'-nitro-N-nitrosoguanidine
MN-PCEs: Micronucleated polychromatic erythrocytes
NCEs: Normochromatic erythrocytes
NIF: Net induction frequency
PCEs: Polychromatic erythrocytes
ROS: Reactive oxygen species.
